# Sporoderm‐broken spores of *Ganoderma lucidum* modulate hepatoblastoma malignancy by regulating RACK1‐mediated autophagy and tumour immunity

**DOI:** 10.1111/jcmm.18223

**Published:** 2024-03-07

**Authors:** Rui Shen, Yang Ge, Yunpeng Qin, Hang Gao, Hongyan Yu, Huazhang Wu, Hang Song

**Affiliations:** ^1^ Graduate School Anhui University of Chinese Medicine Hefei China; ^2^ School of Integrated Chinese and Western Medicine Anhui University of Chinese Medicine Hefei China; ^3^ Anhui Province Key Laboratory of Translational Cancer Research Bengbu Medical College Bengbu China

**Keywords:** *Ganoderma lucidum*, hepatoblastoma, O‐GlcNAcylation, RACK1, sporoderm‐broken spores

## Abstract

Hepatoblastoma (HB), a primary liver tumour, is notorious for its high metastatic potential and poor prognosis. *Ganoderma lucidum*, an edible mushroom species utilized in traditional Chinese medicine for addressing various tumour types, presents an intriguing avenue for HB treatment. However, the effectiveness of *G. lucidum* in managing HB and its underlying molecular mechanism necessitates further exploration. Standard in vitro assays were conducted to evaluate the impact of sporoderm‐broken spores of *G. lucidum* (SBSGL) on the malignant characteristics of HB cells. The mechanism of SBSGL in treating HB and its tumour immunomodulatory effects were explored and validated by various experiments, including immunoprecipitation, Western blotting, mRFP‐GFP‐LC3 adenovirus transfection and co‐localization analysis, as well as verified with in vivo experiments in this regard. The results showed that SBSGL effectively inhibited the malignant traits of HB cells and suppressed the O‐GlcNAcylation of RACK1, thereby reducing its expression. In addition, SBSGL inhibited immune checkpoints and regulated cytokines. In conclusion, SBSGL had immunomodulatory effects and regulated the malignancy and autophagy of HB by regulating the O‐GlcNAcylation of RACK1. These findings suggest that SBSGL holds promise as a potential anticancer drug for HB treatment.

## 1 INTRODUCTION

Hepatoblastoma (HB), accounting for approximately 1% of all paediatric malignancies with an annual incidence of 1.5 cases per 1 million people, has steadily increased over the past three decades.^1^ While the general survival rate for HBs has improved from 30% to 70%, the development's molecular intricacies remain enigmatic.^2^ This perplexing pathogenesis involves a multifaceted interplay of genetic and environmental factors.^3^ Genetic anomalies, particularly on chromosomes 8q and 20q, have been associated with a heightened susceptibility to HBs.^4^ These mutations often coincide with abnormal activation of the Wnt/β‐catenin pathway, potentially fuelling HB development.^5^ On the other hand, the role of environmental factors, such as maternal smoking during pregnancy, remains less clear and necessitates further investigation.^6^ Presently, the most effective treatment for HBs involves surgery, followed by chemotherapy and liver transplantation.^7^ Neoadjuvant chemotherapy, particularly utilizing cisplatin, is employed to shrink tumours and enhance resection rates, albeit with substantial side effects and the risk of drug resistance, severely impacting patients' quality of life.^8^ To elevate the survival prospects of HB patients, there is a pressing need to develop novel drugs targeting the underlying mechanisms driving HB tumorigenesis and progression.

O‐GlcNAcylation, a common post‐translational protein modification, plays a crucial role in cancer cells. Studies have identified several proteins undergoing O‐GlcNAcylation in HB cells, such as HSPB1, which, when modified, enhances cell proliferation and chemoresistance.^9^ Moreover, O‐GlcNAc transferase (OGT), the enzyme responsible for catalysing this modification, upregulates the oncogene miR‐483‐3p in HB cells, suggesting that alterations in O‐GlcNAc activation can significantly influence HB progression.^10^ Activated C‐kinase receptor 1 (RACK1), a tryptophan‐aspartate repeat protein family member, is involved in vital physiological functions, including cell growth, gene transcription and protein synthesis.^11^ In various cancer types, such as oral squamous cell carcinoma, gastric cancer and cervical cancer, RACK1 has emerged as a critical regulatory gene.^12^ Furthermore, O‐GlcNAcylation of RACK1 has been shown to promote tumour development, with elevated RACK1 O‐GlcNAcylation levels detected in hepatocellular carcinoma patient liver samples, correlating with tumour proliferation and recurrence.^13^ However, no direct links between RACK1 and HB development or progression have been established to date. Given the importance of O‐GlcNAcylation in regulating HB development, understanding how this post‐translational modification influences HB development is of utmost significance.

*Ganoderma lucidum* (Leyss. ex Fr.) Karst, a therapeutic fungus with a centuries‐old tradition of use in China, is renowned for its health‐enhancing properties. It boasts an array of biological benefits, including immunomodulation and anticancer potential, making it a promising candidate for addressing various conditions, such as high blood pressure, diabetes, colitis and cancer.^14^, ^15^, ^16^, ^17^, ^18^ Several components of *G. lucidum*, including polysaccharides, triterpenoids, fatty acids, steroids, alkaloids and lactones, have been suggested as contributors to its therapeutic efficacy.^19^, ^20^ *Ganoderma* spore powder, the seed of *G. lucidum*, is a minute oval germ cell expelled during the fungus's growth and maturation. With their hard chitin cellulose double‐walled structure, these spores present challenges for human absorption. However, following cell wall lysis, the resulting sporoderm‐broken spores of *G. lucidum* (SBSGL) exhibit improved suitability for gastrointestinal absorption, with triterpenoids and polysaccharides being the predominant components.^21^ While research has highlighted the anti‐tumour effects of polysaccharides extracted from SBSGL and their role in inhibiting malignant tumour progression in conditions like gastric cancer and osteosarcoma, limited investigation has been conducted on the impact of SBSGL on HB.^22^, ^23^

In light of our hypothesis linking RACK1 O‐GlcNAcylation to HB progression, we conducted in vitro and in vivo assessments of the effect of SBSGL on HB cells. Our findings suggest that SBSGL is a potential agent for tumour immunotherapy and modulates the malignancy and autophagy of HB by regulating the O‐GlcNAcylation of RACK1.

## 2 MATERIALS AND METHODS

### 2.1 Cell culture

HepG2 (ATCC, lot no. HB‐8065) and Huh6 (Wuhan Sunn Technology Co., lot no. SNL‐084) human HB cell lines were cultured in Dulbecco's modified Eagle medium (DMEM, Hyclone, lot no. SH30022.01) supplemented with 10% fetal bovine serum (Biological Industries, lot no. 04‐001‐1A) and 1% penicillin–streptomycin (Shanghai Biyuntian Biotechnology Co., lot no. C0222). Cells were maintained at 37°C in a 5% CO_2_ incubator (Shanghai Lishen Scientific Instruments Co., Ltd., China, HF160W), and the culture medium was refreshed every other day.

### 2.2 SBSGL preparation

*Ganoderma lucidum* spore powder was sourced from HuangshanYunle *Ganoderma Lucidum* Co., Ltd. (lot no. 20220829‐3‐12). Spores were incubated in DMEM at 37°C for 30 min in a water bath, followed by sonication for 60 min at 25°C, 300 W and 40 kHz. After centrifugation for 2 min at 4000 rpm, the supernatant was collected and stored at −20°C.

### 2.3 Cell viability assays

Cells were seeded in 96‐well plates at a 3000 cells/well density and allowed to attach overnight. The culture medium was then replaced with fresh medium containing different concentrations of SBSGL, while control cells received SBSGL‐free medium. After 24, 48 and 72 h of incubation at 37°C, 10 μL of CCK8 reagent (GLPBIO, lot no. GK10001) was added to each well. Following a 4‐hour incubation with continuous shaking, the absorbance was measured at 490 nm using an enzyme labeler (Shanghai Ganmin Analytical Instruments Co., Ltd., China, 318C+). Cell viability was calculated using the formula: Cell viability (%) = (OD drug treatment group—OD blank group)/(OD non‐drug group—OD blank group) × 100%. Each experiment was performed in quadruplicate.

### 2.4 Apoptosis assay

Cells were seeded in six‐well plates at 1 × 10^5^ cells/mL density and treated with SBSGL at 0, 600, 1200 and 1800 μg/mL concentrations for 48 h. After trypsinization without EDTA, the cells were washed with phosphate‐buffered saline (PBS), resuspended at a density of 1 × 10^6^ cells/mL, and incubated with 5 μL Annexin V‐FITC (Shanghai Biyuntian Biotechnology Co., lot no. C1062S) for 15 min, followed by 5 μL propidium iodide (PI) staining solution (Merck, lot no. P4864‐10ML) for 5 min in the dark at room temperature. Flow cytometry (Aison Biological Co., Ltd., China, NovoCyte 2060R) was used to determine the apoptosis rate.

### 2.5 Transwell assay

Cells were treated with SBSGL at 0, 600, 1200 and 1800 μg/mL concentrations and incubated for 48 h. Transwell in 24‐well plates (Thermo Fisher Scientific, lot no. 140644) were employed for the transwell assay. Cells in serum‐free medium were added to the upper chamber at a 2.5 × 10^5^ cells/mL density, while the lower chamber contained culture medium. After 48 h of incubation under normal conditions, the chambers were separated and washed with PBS, and the cells on the lower surface were fixed with 4% formaldehyde (Biosharp, lot no. 1912A05) for 15 min. Staining with 700 μL crystal violet (Biyuntian Bio‐Technology Co., lot no. C0121) for 30 min followed. After washing with PBS, cell counting was performed under a microscope.

### 2.6 Wound healing assay

Cells were grown to confluence in six‐well plates, and a sterile pipette tip was used to create a scratch. The monolayer was washed three times with PBS to remove detached cells, and a fresh medium containing 0, 600, 1200 and 1800 μg/mL concentrations of SBSGL was added. The wound area was photographed at 0 and 48 h of culture, and changes in the scratch area were measured to assess cell migration rate. Each concentration and the blank control were tested in triplicate simultaneously.

### 2.7 Animal study

Animal experiments were performed as described previously.^24^ Male BALB/c nude mice and BALB/c mice were purchased from Beijing Vital River Laboratory Animal Technology Co., Ltd. and were acclimatized for 1 week before the experiment.

BALB/c nude mice were injected subcutaneously with 1 × 10^7^ HepG2 cells, and tumours were excised when the tumour volume reached 100 mm^3^. Fresh tumour samples from the BALB/c nude mouse tumour model were cut into 2 mm × 2 mm × 2 mm. After BALB/c mice were intraperitoneally injected with cyclophosphamide (50 mg/kg) for three consecutive days, the tumour cut pieces were implanted into their right dorsum. When the tumours reached a certain volume, the BALB/c mice were randomly divided into four groups of six mice each and were gavaged with different concentrations of SBSGL (0, 75, 150, 300 mg/kg) for 28 consecutive days. In order to avoid the recovery of immune function in mice, cyclophosphamide (50 mg/kg) was injected once a week. After the mice were euthanized, tumours, spleens and blood were collected. Tumour volume = 0.5 × length × width^2^.

### 2.8 Western blotting

Cells were cultured in six‐well plates with SBSGL at 0, 600, 1200 and 1800 μg/mL concentrations. Radio immunoprecipitation assay buffer (Shanghai Biyuntian Biotechnology Co., lot no. P0013C) with a protease inhibitor was used to extract proteins. Equal amounts of protein from each sample were transferred to polyvinylidene fluoride membranes (Merck, lot no. IPFL00010). Membranes were incubated with primary antibodies against RACK1 (Cell Signaling Technology, lot no. 5432, 1:1000), OGT (Cell Signaling Technology, lot no. 24083, 1:1000), O‐GlcNAc (Thermo Fisher Scientific, lot no. MA1‐072, 1:1000), LC3B (Cell Signaling Technology, lot no. 3868, 1:1000), p62 (Cell Signaling Technology, lot no. 5114, 1:1000), PD‐L1 (Cell Signaling Technology, lot no. 13684, 1:1000), Myc‐tag (Cell Signaling Technology, lot no. 2276, 1:1000) and glyceraldehyde‐3‐phosphate dehydrogenase (GAPDH, Cell Signaling Technology, lot no. 5174, 1:1000) overnight at 4°C. After washing with Tris‐buffered saline with Tween (TBST, Biosharp, lot no. 21259419), secondary antibodies goat anti‐rabbit IgG‐HRP (Abbkine, lot no. A21020, 1:10,000) and goat anti‐mouse IgG‐HRP (Abbkine, lot no. A21010, 1:10,000) were applied for 1 h at room temperature. Membranes were washed thrice with TBST, and visualization was achieved using an ECL luminescence kit (Shanghai Biyuntian Biotechnology Co., lot no. P0018S).

### 2.9 Immunofluorescence

For immunofluorescence assays, cells were treated with a medium dose of SBSGL for 48 h, washed thrice with cold PBS, fixed overnight with 4% paraformaldehyde and washed thrice with 0.5% Triton for 5 min each. After blocking with 20% goat serum in BSA for 1 h at room temperature, the cells were sequentially incubated with primary and secondary antibodies. After three additional PBS washes, cells were counterstained with 2‐(4‐amidinophenyl)‐6‐indolecarbamidine dihydrochloride (DAPI, Biyuntian Bio‐Technology Co., lot no. C1002) for 5 min, washed once with PBST for 10 min and twice more for 10 min each. The slides were sealed using an anti‐fade mounting medium and examined under a fluorescence microscope.

### 2.10 UPLC‐Q‐TOF‐MS/MS analysis

A 0.4 g sample of SBSGL powder was suspended in 4 mL of water and incubated at 37°C for 30 min, followed by sonication for 30 min at 25°C. After centrifugation at 4000 rpm for 2 min, the supernatant was collected. Ultra‐high‐performance liquid chromatography (UHPLC) was conducted using an Agilent 5 TC‐C18(2) column (250 × 4.6 mm, 5 μm; Agilent, USA), with a detection wavelength of 220 nm, a flow rate of 1 mL/min and a column temperature of 30°C. A 10 μL aliquot of the supernatant was injected into the UHPLC, and components were eluted according to a gradient program: 0–20 min, 5%–25% A; 20–28 min, 25%–55% A; 28–55 min. Electrospray ionization in positive‐ion mode was used for mass spectrometry analysis. The atomizer temperature was set at 300°C, the gas flow rate was 8 L/min, the spray pressure was 30 psi, the collision voltage was 135 V, the capillary voltage was 3500 V, the cone voltage was 65 V, and the mass range was 50–1700 *m/z*. The Masshunter software was used for component identification based on molecular and fragment ion information.

### 2.11 Immunoprecipitation assays

Experiments were performed according to the instructions of the Pierce™ Classic Magnetic IP/Co‐IP kit (Thermo Fisher Scientific, lot no. 88804). Cells were incubated in pre‐cooled IP Lysis Buffer at 4°C for 5 min, and then the lysate was centrifuged at 13,000 × *g* for 10 min to collect the supernatant. The corresponding immunoprecipitating antibody was added to the cell lysate containing 500 μg of protein, equilibrated to 500 μL with IP lysis buffer and incubated overnight at 4°C. The next day, the antibody–protein complexes were extracted with magnetic beads and subjected to western blot experiments.

### 2.12 mRFP‐GFP‐LC3 adenovirus transfection and co‐localization analysis

Cells were cultured in DMEM until reaching semi‐confluence and then transfected with mRFP‐GFP‐LC3 for 6 h at room temperature. After two PBS washes, cells were incubated in a culture medium for 24 h and then treated with SBSGL at concentrations of 0, 600, 1200 and 1800 μg/mL for an additional 48 h.

### 2.13 ELISA

The cytokines TNF‐α, IL‐2, IL‐10 and IL‐6 levels were determined separately in the serum of mice collected from animal experiments according to the instructions for the ELISA kit. The kits were purchased from YoBiBiotech Co., Ltd., and the batch numbers were U96‐3112E, U96‐1498E, U96‐1517E and U96‐1511E.

### 2.14 Statistical analysis

All data were analysed using GraphPad Prism (version 8.0) software. The *t*‐test was used for two‐group comparisons, while one‐way analysis of variance (ANOVA) was employed for multiple‐group comparisons. A significance level of *p* < 0.05 was considered statistically significant.

## 3 RESULTS

### 3.1 Chemical composition of SBSGL

**FIGURE 1:**
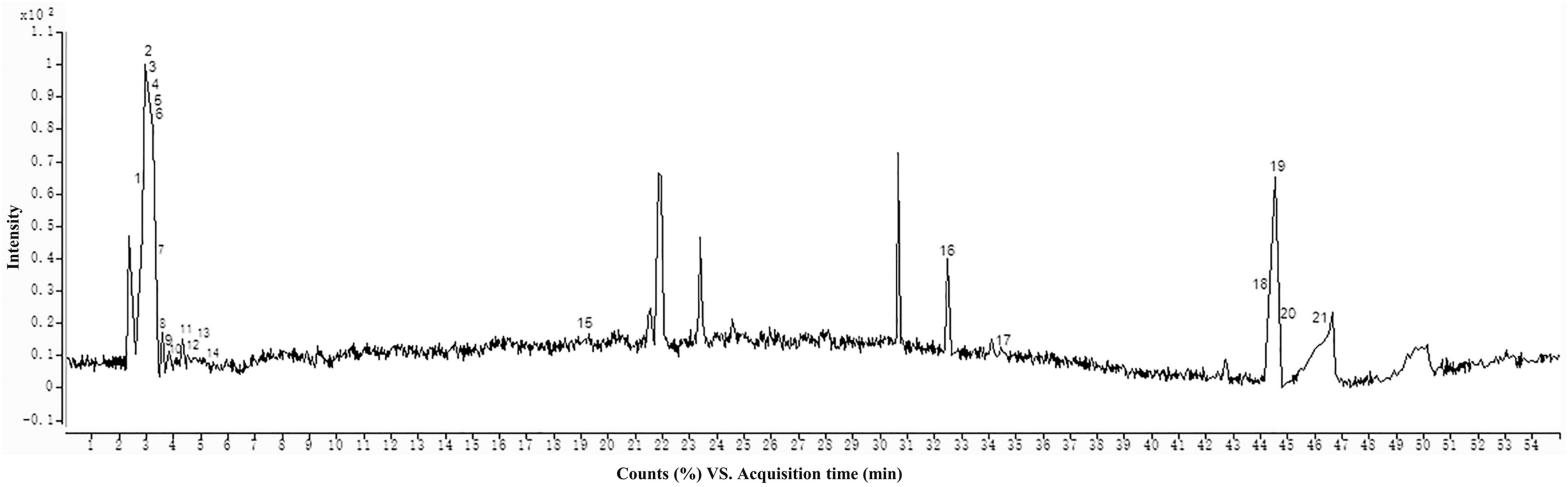
UPLC‐Q‐TOF‐MS/MS analysis of SBSGL. Following centrifugation of the spore powder in water, the supernatant was subjected to chromatographic analysis and mass spectrometry. Total ion flow maps were collected in negative ion mode.

We analysed the chemical composition of SBSGL using UPLC‐Q‐TOF‐MS/MS in ESL positive‐ion mode. The chromatograms in Figure 1 illustrate the chemical constituents present in SBSGL. These constituents include polysaccharides, triterpenes and adenosine, such as Cellobiose, Pyroglutamic acid, 5′‐Adenosine monophosphate and other specific components. Please refer to Table 1 for detailed information.

**TABLE 1:** Chemical constituents identified in SBSGL based on UPLC‐Q‐TOF‐MS/MS.

No	*t*_R_/min	Compound name	Ion species	Molecular formula	Theoretical value (*m/z*)	Measured value (*m/z*)	Deviation	Fragment ion (*m/z*)
1	2.97	Securinol A	[M + Na]+	C_13_H_17_NO_3_	235.28	235.12	−1.17	258.11, 184.07, 166.06, 71.07
2	3.00	Dulcitol	[M + Na]+	C_6_H_14_O_6_	182.17	182.08	−0.08	205.07, 183.09, 147.07, 129.06
3	3.13	(+)‐1‐Hydroxypinoresinol‐4′‐O‐Beta‐d‐glucopyranoside	[M + H]+	C_26_H_32_O_12_	536.50	536.19	3.55	537.20, 325.11, 235.08, 125.00
4	3.20	Cellobiose	[M + Na]+	C_12_H_22_O_11_	342.30	342.12	−3.75	365.11, 221.02, 205.07, 147.07
5	3.23	Boschniakine	[M + Na]+	C_10_H_11_NO	161.20	161.08	0.62	184.07, 162.11, 146.12, 118.12
6	3.30	Erycibelline	[M + H]+	C_7_H_13_NO_2_	143.18	143.09	−1.59	144.10, 124.04, 113.06, 108.05
7	3.66	Histamine	[M + H]+	C_5_H_9_N_3_	111.15	111.08	−2.88	112.09, 111.04, 109.10, 101.66
8	3.79	5′‐Adenosine monophosphate	[M + H]+	C_10_H_14_N_5_O_7_P	347.22	347.06	−1.1	348.07, 136.06, 119.04, 109.10
9	3.80	Gamma‐Guanidinobutyric acid	[M + H]+	C_5_H_11_N_3_O_2_	145.16	145.09	−0.4	146.09, 129.05, 128.08, 111.05
10	4.29	Nicotinic acid	[M + H]+	C_6_H_5_NO_2_	123.11	123.03	0.79	124.04, 106.03, 80.05, 78.03
11	4.35	Nicotinamide	[M + H]+	C_6_H_6_N_2_O	122.12	122.05	0.53	123.06, 96.04, 80.05, 78.04
12	4.74	D‐threo‐Isocitric acid	[M + Na]+	C_6_H_8_O_7_	192.12	192.03	−0.27	215.02, 193.04, 152.03, 147.03
13	4.81	Pyroglutamic acid	[M + H]+	C_5_H_7_NO_3_	129.11	129.04	−2.88	130.05, 85.05, 84.05, 56.05
14	5.26	Adenosine	[M + H]+	C_10_H_13_N_5_O_4_	267.24	267.10	−1.58	268.10, 136.06, 119.04, 92.02
15	19.07	Solanocapsine	[M + Na]+	C_27_H_46_N_2_O_2_	430.70	430.36	0.84	453.34, 413.27, 376.65, 322.25
16	32.45	4′‐O‐beta‐Glucopyranosyl‐5‐O‐methylvisamminol	[M + Na]+	C_22_H_28_O_10_	452.50	452.17	−0.9	475.16, 305.25, 291.23, 209.11
17	34.43	Phthalic anhydride	[M + H]+	C_8_H_4_O_3_	148.11	148.02	0.4	149.02, 122.10, 121.05, 93.03
18	44.33	Ditertbutyl phthalate	[M + Na]+	C_16_H_22_O_4_	278.34	278.15	−0.68	301.14, 279.16, 150.03, 104.99
19	44.53	3‐Butylidene‐7‐hydroxyphthalide	[M + H]+	C_12_H_12_O_3_	204.22	204.08	−0.34	205.09, 149.02, 65.04, 93.03
20	44.66	Cucurbitacin E	[M + Na]+	C_32_H_44_O_8_	556.70	556.30	−0.48	579.29, 303.15, 141.11, 95.09
21	46.27	Huratoxin	[M + Na]+	C_34_H_48_O_8_	584.70	584.34	−2.1	607.82, 568.38, 557.45, 456.81

### 3.2 SBSGL suppresses the proliferation and migration of HB in vitro

**FIGURE 2:**
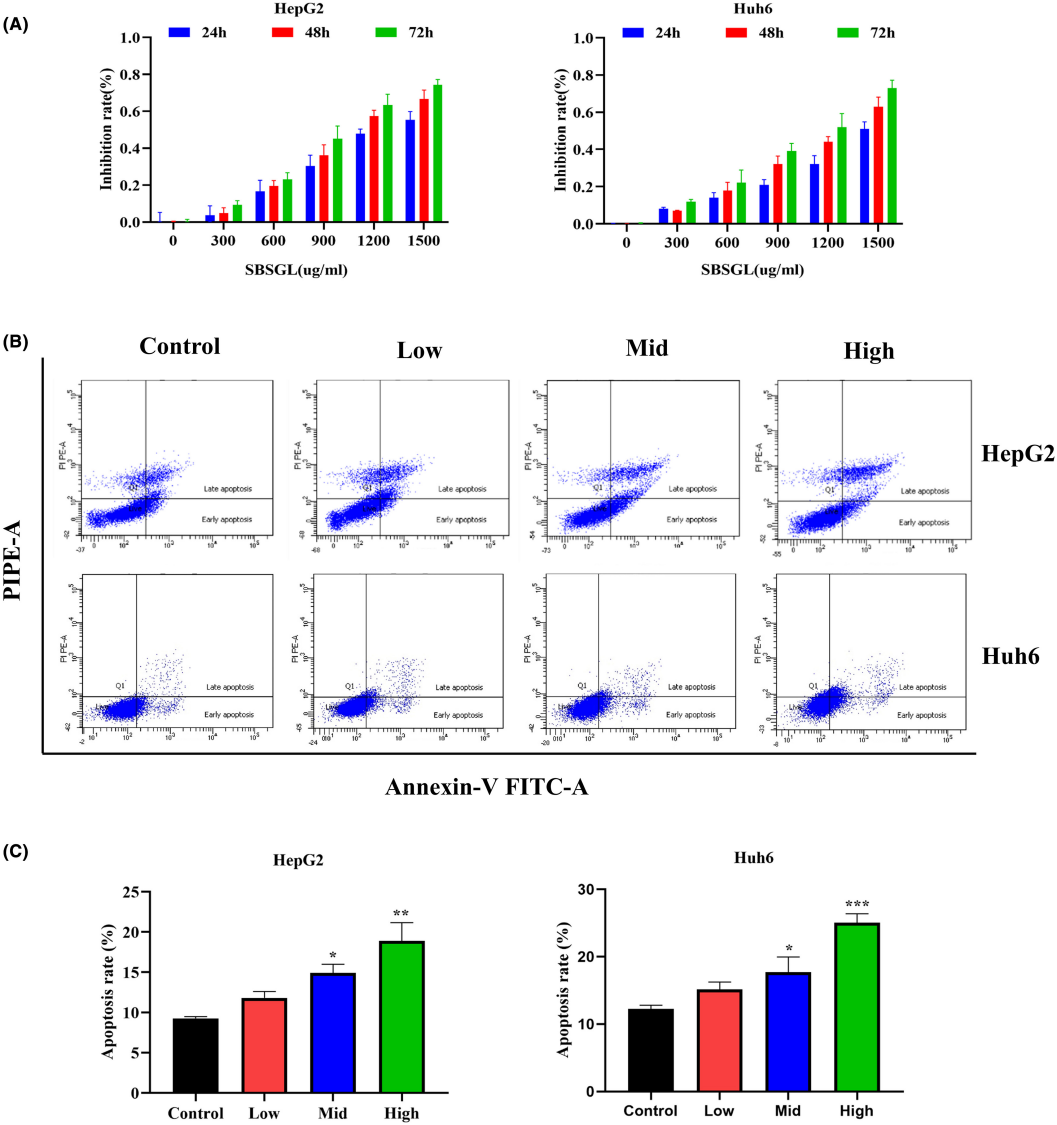
SBSGL suppresses the malignant phenotype of HB. (A) Inhibition rates of HepG2 and Huh6 cells treated with SBSGL for 24, 48, and 72 h. (B, C) Apoptosis of HepG2 and Huh6 cells and corresponding quantitative analysis. *p < 0.05, **p < 0.01, ***p < 0.001. (The same applies below).

**FIGURE 3:**
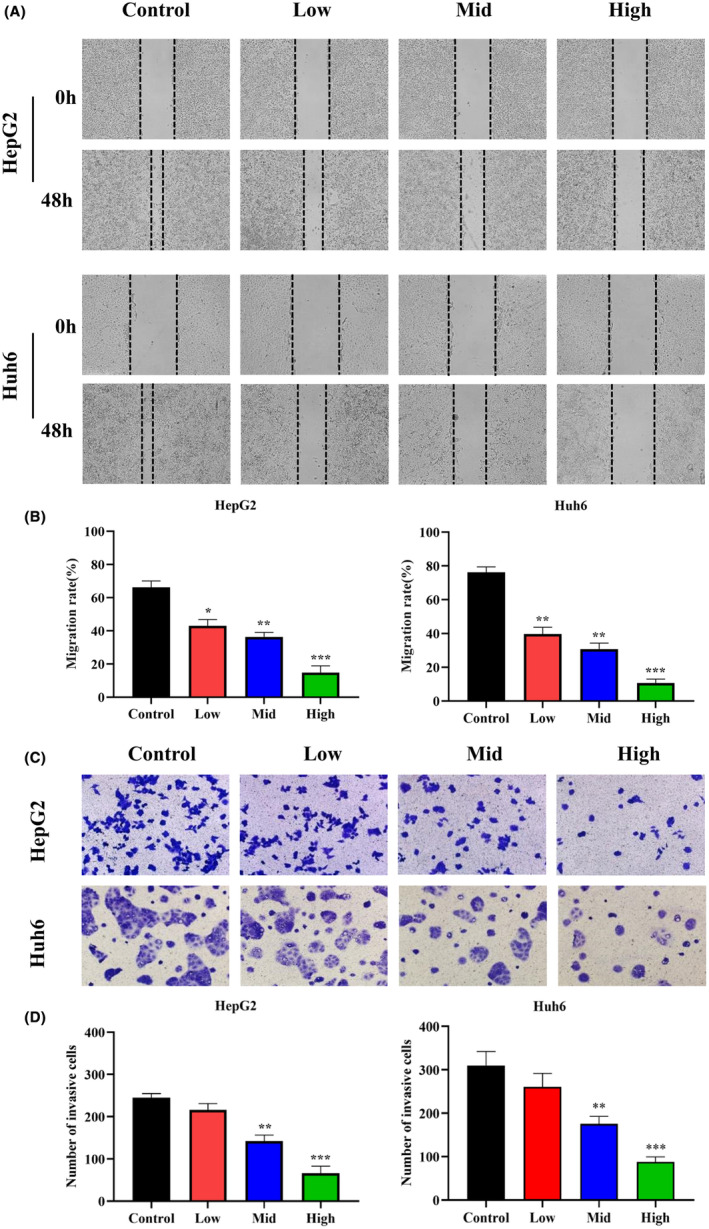
Inhibition of HepG2 and Huh6 migration by SBSGL in vitro. (A, B) Cell scratch assay showed that SBSGL significantly inhibits HB cell migration with increasing SBSGL concentration. (C, D) Transwell results show SBSGL's ability to inhibit the invasive capacity of HB cells in vitro. Images captured at ×200.

HB cells were treated with various concentrations of SBSGL (0, 300, 600, 900, 1200, 1500 μg/mL), and cell viability was assessed using the CCK‐8 method at 24, 48 and 72 h (Figure 2A). The IC50 values for HepG2 cells at 24, 48 and 72 h were 1311, 1102 and 964.3 μg/mL, respectively. For Huh6 cells, the IC50 values at the same time points were 1592, 1252 and 1069 μg/mL. After careful consideration, we selected the 48‐h time point for further analysis, as it showed a reasonable balance between the two cell lines. The average IC50 values for both cell lines at 48 h were approximately 1200 μg/mL, and we established low, medium and high concentration groups of SBSGL according to 50%, 100% and 150% of this value. The apoptosis assay results revealed a significant increase in the proportion of HB cells in the early and late stages of apoptosis with increasing SBSGL concentration (Figure 2B,C). Moreover, SBSGL effectively inhibited the migration of both HepG2 and Huh6 cells, as demonstrated by wound healing and transwell assays (Figure 3A–D).

### 3.3 SBSGL inhibits the O‐GlcNAcylation of RACK1 protein

**FIGURE 4:**
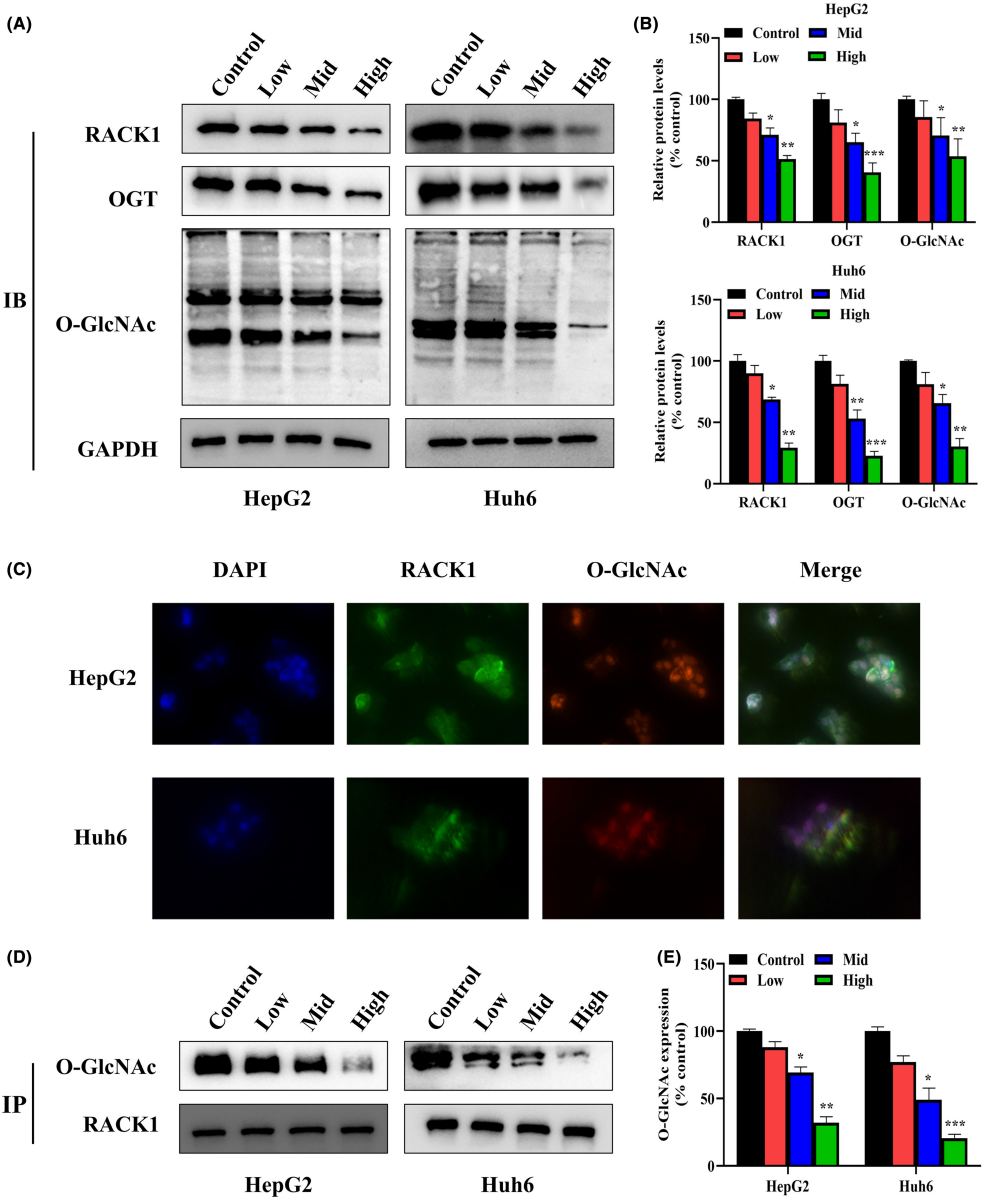
SBSGL reduces the expression of OGT and RACK1 proteins in HB cells. (A, B) Western blot analysis reveals decreased expression levels of OGT, RACK1 and O‐GlcNAcylation proteins in treated cells. (C) Immunofluorescence results demonstrate typical intracellular localization of RACK1 and O‐GlcNAcylation. (D, E) IP results indicate a gradual reduction in the expression of O‐GlcNAc modification of RACK1 with increasing SBSGL concentration.

The levels of RACK1, OGT and total O‐GlcNAcylation were significantly reduced in both HB cells after SBSGL treatment compared to the control (Figure 4A,B). In addition, Immunofluorescence result demonstrated the co‐localization between RACK1 and O‐GlcNAcylation in HB cells (Figure 4C). Finally, immunoprecipitation results indicate that SBSGL suppressed O‐GlcNAcylation of RACK1 in HB cells (Figure 4D,E).

### 3.4 SBSGL diminishes RACK1 protein expression by inhibiting O‐GlcNAc modification

**FIGURE 5:**
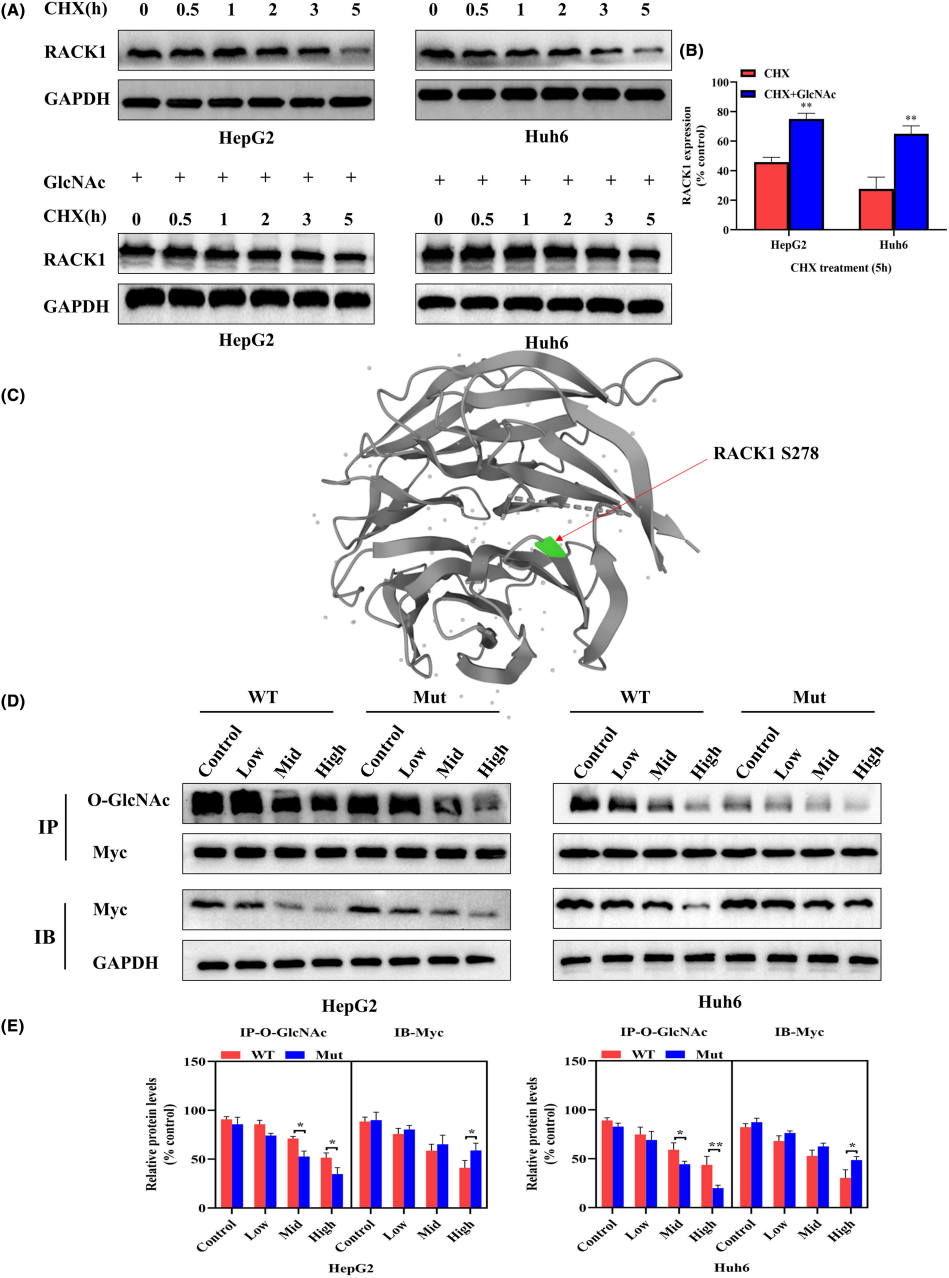
SBSGL diminishes RACK1 protein expression by inhibiting O‐GlcNAc modification. (A, B) Western blot results illustrate the correlation between RACK1 protein degradation and O‐GlcNAcylation. (C) Prediction of the most likely RACK1 modification site, RACK1 S278, using the O‐GlcNAc site predictor (https://services.healthtech.dtu.dk/service.php?NetOGlyc‐4.0). (D, E) IP results demonstrate a more significant decrease in O‐GlcNAcylation expression levels in the Mutant group (mutation of Serine to Alanine) compared to the wild‐type group after SBSGL treatment. The IB results reveal a more rapid decrease in RACK1 protein expression in the wild‐type group with increasing SBSGL concentration.

HB cells were treated with the inhibitor of protein synthesis, cycloheximide (CHX, 50 μg/mL), for 5 h. The half‐life experiments showed higher levels of RACK1 protein expression in GlcNAc‐treated substrates compared with the controls, suggesting that RACK1 degradation was associated with O‐GlcNAcylation modification of RACK1 (Figure 5A,B). Then we downloaded the Rack1 protein sequence in the UniProt database (https://www.uniprot.org/) and entered the protein sequence on the web page (https://services.healthtech.dtu.dk/service.php?NetOGlyc‐4.0) for prediction. Our prediction indicated that S278 was the most likely modification site of RACK1 (Figure 5C). To confirm this, we conducted site‐directed mutagenesis to generate a Myc‐tagged mutant S278 RACK1 protein (mutation of Serine to Alanine) overexpressed in cells. The immunoprecipitation assay revealed that the mutants expressed more RACK1 proteins than wild‐types as SBSGL concentrations increased, indicating that SBSGL inhibited the O‐GlcNAcylation of S278 in the RACK1 protein and reduced its protein levels (Figure 5D,E).

### 3.5 SBSGL induces autophagy and inhibits autophagic fluxes

It was found that the knockdown of RACK1 induced LC3‐dependent non‐classical autophagy.^25^ In addition, a study on autophagy showed that FGF21 upregulation of RACK1 induced autophagy and inhibited atherosclerosis.^26^ RACK1 is upregulated in a variety of human cancers and has been suggested to contribute to the development and progression of human cancers. An experiment on colon cancer revealed that RACK1‐induced autophagy promoted the proliferation of colon cancer cells. Thus, the autophagy flow experiment was conducted.^27^ Therefore, we hypothesize SBSGL may regulate the autophagy of HB cells.

**FIGURE 6:**
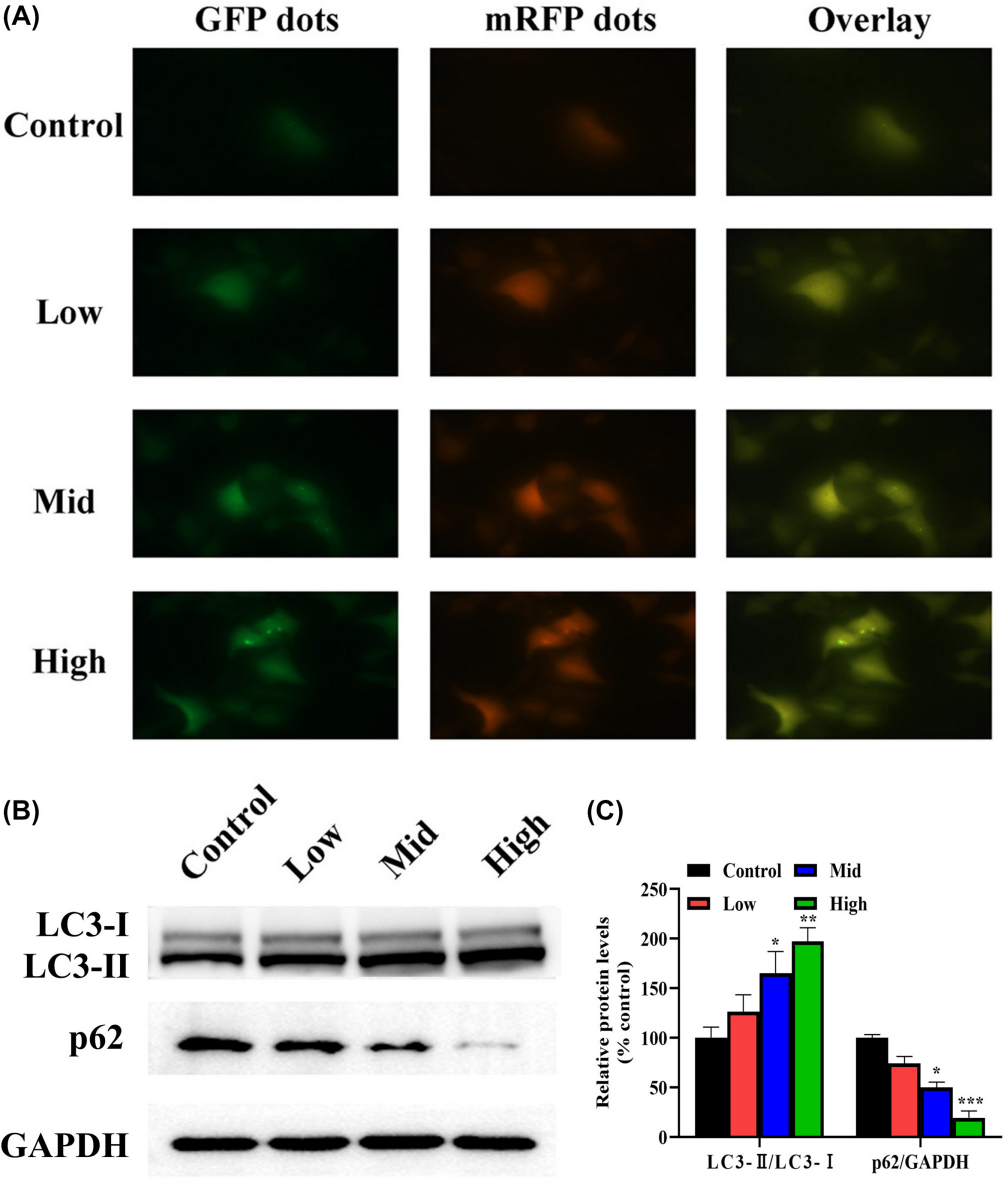
SBSGL induces autophagy and inhibits autophagic flux. (A) Fluorescence microscopy reveals co‐localization analysis, with yellow puncta indicating autophagosomes and red puncta representing autolysosomes. Scale bar, 20 μm. (B, C) Western blotting indicates an increase in LC3‐II/LC3‐I ratio and a decrease in p62 expression in the SBSGL treatment group.

SBSGL was found to affect autophagosome‐lysosome fusion, a crucial step in autophagy. The number of yellow puncta in merged images progressively increased with increasing SBSGL concentration (600–1800 μg/mL), indicating that SBSGL impeded autophagosome‐lysosome fusion, resulting in impaired autophagic flux in HepG2 cells (Figure 6A). Western blotting further supported this by showing increased LC3‐II/LC3‐I ratios with higher SBSGL concentrations while p62 expression decreased accordingly (Figure 6B,C).

### 3.6 SBSGL inhibits the growth of HB in vivo and modulates immune function

The transmembrane protein PD‐L1 expressed on the tumour surface can bind to PD‐1 expressed on immune cells, its overexpression can suppress the anti‐tumour immune response of T cells, resulting in the escape of tumour cells from the immune system.^28^ In addition, altering the concentration of cytokines in the tumour immune microenvironment modulates the anti‐tumour immune response. Among them, TNF‐α and IL‐2 are produced by Th1 cells and mediate anti‐tumour effects, while Th2 cells produce IL‐6 and IL‐10 and promote tumour growth by suppressing the immune system.^29^ Since RACK1 expression is associated with immune cell function as well as cytokine production and has a dual role in immunity and cancer,^30^ we performed relevant animal and immune experiments, including the detection of PD‐1/L1 immune checkpoints and cytokines.

**FIGURE 7:**
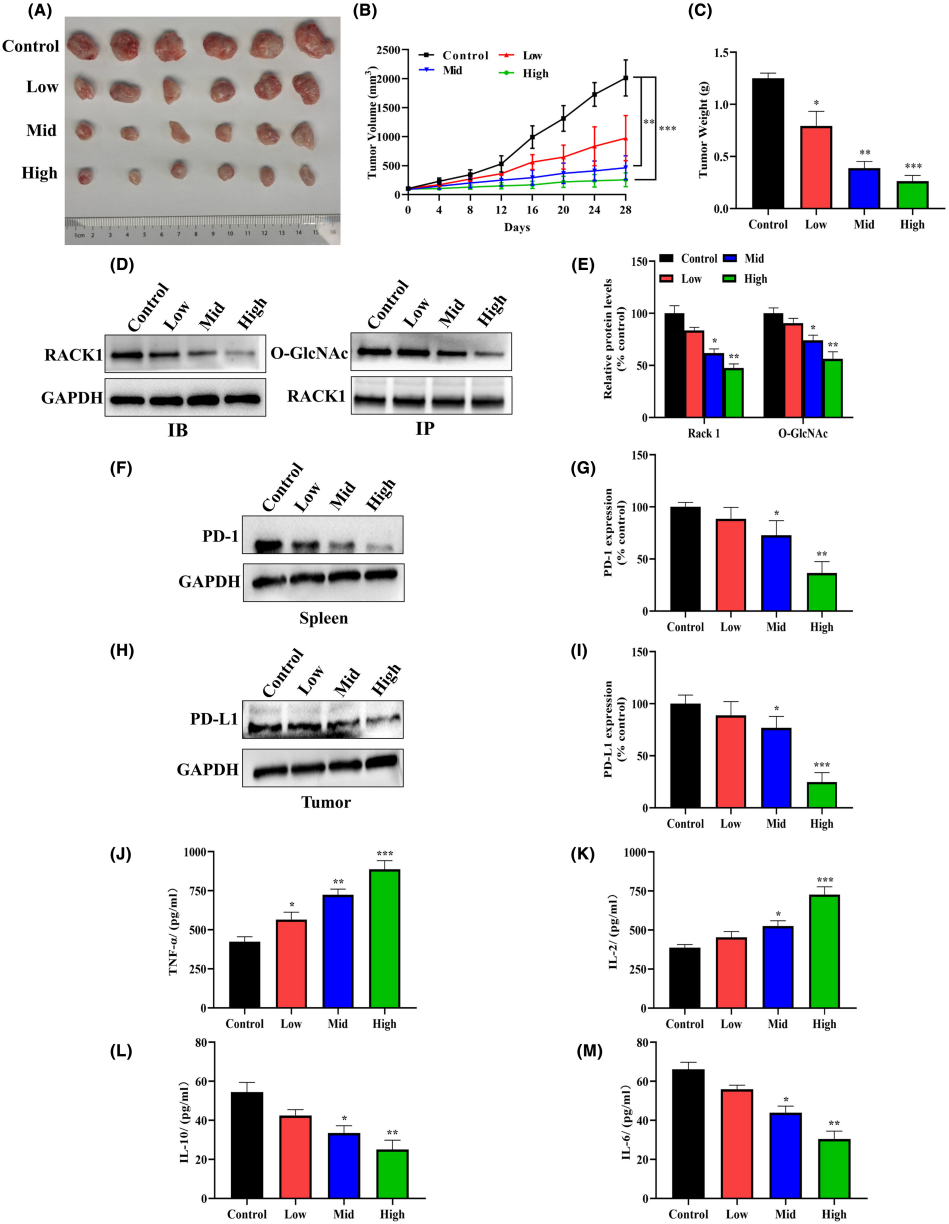
SBSGL inhibits the growth of HB in vivo and modulates immune function. (A) Photographs of excised tumours in BALB/c mice. (B) Tumour volume. (C) Tumour weight. (D, E) As SBSGL concentration increased, both RACK1 protein expression and its O‐GlcNAcylation level were reduced. SBSGL down‐regulates PD‐1 protein expression in spleen (F, G) and PD‐L1 protein in tumour tissues (H, I). Compared with the control group, TNF‐α (J) and IL‐2 (K) levels increased in a concentration‐dependent manner in the SBSGL‐treated group, while IL‐10 (L) and IL‐6 (M) levels showed the opposite trend.

The in vivo effects of SBSGL were evaluated in a subcutaneous tumour model in BALB/c mice. Tumours were treated with different doses of SBSGL for 28 days after reaching the appropriate size. As shown in Figure 7A,C, mice treated with low, medium, and high doses of SBSGL had smaller tumour volume and lighter tumour weight than controls, with the highest dose having the most significant effect. In addition, we extracted tumour tissue proteins for experiments, and the results showed that SBSGL inhibited the O‐GlcNAcylation of RACK1 protein and reduced its expression level, validating the results of the cellular experiments (Figure 7D,E).

In addition, SBSGL could down‐regulate the expression of PD‐1 protein in spleen (Figure 7F,G) and PD‐L1 protein in tumour tissues (Figure 7H,I). Compared with the control group, with the increase in drug concentration, TNF‐α and IL‐2 contents in the SBSGL‐treated group were increased, and there was a tendency to decrease IL‐10 and IL‐6 contents (Figure 7J–M). These results suggest that SBSGL may play a tumour‐suppressive role by inhibiting immune checkpoints and regulating the cytokine TNF‐α and IL‐2 pathways to activate Th1‐type cells.

## 4 DISCUSSION

SBSGL, known for its anti‐tumour and immunomodulatory properties, has been studied extensively in various contexts. Prior research has indicated that SBSGL can suppress angiogenesis and cellular autophagy, making it a promising candidate for halting osteosarcoma progression.^22^ Its immunomodulatory functions, particularly its influence on intestinal flora, have been well‐documented.^21^ In addition, SBSGL can potentially reduce oxidative stress and prevent cadmium (II)‐induced hepatotoxicity.^31^ UPLC‐Q‐TOF‐MS/MS analysis showed that the chemical composition in SBSGL is complex, and its functional constituents (e.g., triterpenoids and polysaccharides) exhibit efficacy in detoxification, hepatoprotective and anti‐tumour activities with the treatment of malignant tumours, such as breast and colon cancer, being with documented success.^32^, ^33^, ^34^ Moreover, numerous studies have demonstrated significant antioxidative and anti‐tumour effects of polysaccharides extracted from *G. lucidum*, the main component of SBSGL.^34^, ^35^

The results presented in this study underscore the effectiveness of SBSGL in suppressing the malignant characteristics of HB cells. Specifically, SBSGL impedes HB tumour growth by inhibiting the O‐GlcNAcylation of RACK1, which is involved in multiple signalling pathways, affecting processes such as cell growth and migration and is aberrantly expressed in a wide range of malignant tumours.^36^ Notably, O‐GlcNAcylation is also closely associated with the malignant phenotype of tumours, with a recent study highlighting the critical role of O‐GlcNAcylation in influencing tumour cell proliferation, metastasis, and angiogenesis.^37^ Furthermore, elevated O‐GlcNAcylation levels have been observed in breast cancer tissues, impacting intercellular adhesion and promoting migration and invasion.^38^ Interestingly, similar to the present study, it has been shown that O‐GlcNAcylation modification of RACK1 is associated with tumour progression. For example, increased RACK1 O‐GlcNAcylation was detected in patients with hepatocellular carcinoma, which was associated with poor prognosis after chemotherapy.^13^ In another study, circVPRBP was shown to bind to RACK1 to inhibit its O‐GlcNAcylation modification at the S122 site, which reduced its stability and inhibited lymph node metastasis in cervical cancer.^39^ Studies like these emphasize the significant influence of RACK1 expression and O‐GlcNAcylation modifications on tumorigenesis and metastasis. On the other hand, SBSGL inhibits immune checkpoints and regulates cytokines. Interestingly, RACK1 is also associated with tumour immunotherapy. In oral squamous cell carcinoma, RACK1 promotes M2‐like macrophage polarization, which together with the M2/M1 ratio, predicts poor tumour prognosis.^40^ Moreover, RACK1 promotes autophagy and is a critical regulator of T‐cell homeostasis.^41^ Similar studies have shown that SBSGL is effective in restoring cytotoxic T cells and suppressing immune checkpoints in in vivo and in vitro experiments in breast cancer.^42^ These experiments support the conclusion that SBSGL has an immunomodulatory role and regulates HB malignancy and autophagy by modulating the O‐GlcNAcylation of RACK1.

HB is traditionally treated with chemotherapeutic agents like cisplatin, carboplatin, etoposide, vincristine, irinotecan, and doxorubicin.^8^ While these treatments are effective against cancer, they often lead to severe side effects and toxicity in patients. For example, cisplatin treatment has been associated with hearing loss in over 50% of children with HB.^43^ Traditional Chinese medicine (TCM) has gained attention for its potential role in treating HB. Studies have shown that TCM formulations like Babao Dan can reduce Wnt target gene expression, limit the presence of cancer stem cells, and impair Wnt activation in HB tumours.^44^ Similarly, SBSGL, a TCM with significant anti‐tumour effects, can be used as an immunomodulator to enhance the immune function of cancer patients. In a clinical trial on 47 colorectal cancer patients, several immune parameters such as IL‐1, IL‐6, IFN‐γ, CD3 and CD4 were altered after 12 weeks of oral administration of *G. lucidum*, suggesting that it may be helpful in modulating tumour immunity.^45^ Notably, another clinical trial showed that *G. lucidum* possesses adequate antioxidant capacity and is non‐toxic to the liver and kidney.^46^ In addition, SBSGL may also assist traditional radiotherapy and chemotherapy treatments in improving cancer patients' quality of survival and prognosis jointly. For example, in a randomized controlled trial, an herbal complex containing *G. lucidum* improved immune function, reduced adverse effects and increased survival in patients receiving chemotherapy and radiotherapy.^47^ Therefore, the use of SBSGL in combination with existing anti‐tumour agents in clinical therapy may provide direction in finding the optimal treatment for HB, and we will further explore the functions of individual components in SBSGL and the specific mechanisms of regulating tumour immunity next.

## 5 CONCLUSION

In summary, this study reveals the mechanism of SBSGL in delaying HB progression and its potential application in tumour immunotherapy. The results indicate that SBSGL effectively curbs HB malignancy and autophagic flux by modulating O‐GlcNAc modifications in the RACK1 protein. In addition, it inhibits immune checkpoints and regulates cytokines. These findings suggest that SBSGL holds promise as a clinical treatment for HB. However, further research, including investigations into SBSGL's interactions with other chemotherapeutic agents, is warranted to explore potential combinatorial therapies.

## AUTHOR CONTRIBUTIONS

**Rui Shen:** Data curation (equal); writing – original draft (equal). **Yang Ge:** Writing – original draft (equal). **Yunpeng Qin:** Data curation (equal). **Hang Gao:** Software (lead). **Hongyan Yu:** Conceptualization (equal). **Huazhang Wu:** Conceptualization (equal). **Hang Song:** Funding acquisition (lead); supervision (lead).

## FUNDING INFORMATION

This work was supported by Excellent Young Scholars Project of the Natural Science Foundation of Anhui Province in China (grant number 2108085Y29) and Anhui Province Key Laboratory of Translational Cancer Research (Bengbu Medical College, grant number KFZZ202205).

## CONFLICT OF INTEREST STATEMENT

The author reports no conflicts of interest in this work.

## Data Availability

The data supporting this study's findings are available from the corresponding author upon reasonable request.
